# Behavioural responses of *Anopheles darlingi* (Diptera: Culicidae) to host odours: insights from vertical and horizontal olfactometers

**DOI:** 10.1590/0074-02760250154

**Published:** 2026-02-13

**Authors:** Thais Costa dos Santos, Moreno Magalhães De Souza Rodrigues, Elis Paula de Almeida Batista, Kelly da Silva Paixão, Álvaro Eduardo Eiras, Gabriel Zorello Laporta, Alexandre de Almeida e Silva

**Affiliations:** 1 Universidade Federal de Rondônia, Departamento de Biologia, Porto Velho, RO, Brasil; 2 Fundação Oswaldo Cruz-Fiocruz, Porto Velho, RO, Brasil; 3 Ministério da Saúde, Brasília, DF, Brasil; 4 Universidade Federal de Minas Gerais, Instituto de Ciências Biológicas, Departamento de Parasitologia, Belo Horizonte, MG, Brasil; 5 Centro Universitário Faculdade de Medicina do ABC, Santo André, SP, Brasil

**Keywords:** Anopheles darling, malaria, mosquito behaviour, olfactometry, host-seeking behaviour

## Abstract

**BACKGROUND:**

*Anopheles darlingi* is the primary vector of malaria in the Americas, particularly in the Amazon, where it thrives in forest margins. This species exhibits considerable flexibility in feeding behaviour, adapting to environmental conditions and host availability. Previous studies on its attraction to human odour have relied mainly on baited traps, with limited research using vertical olfactometry to explore host-seeking behaviour.

**OBJECTIVES:**

This study aimed to assess the feasibility of using vertical and horizontal olfactometry to investigate the behavioural responses of both wild and laboratory-reared (F1) *An. darlingi* females to human odours. The odours were presented through direct (hands and feet) and indirect (sweat-impregnated synthetic substrates) stimuli.

**METHODS:**

Wild mosquitoes were collected from Porto Velho and Candeias do Jamari, Brazil, and laboratory-reared (F1) mosquitoes were bred under controlled conditions. A vertical olfactometer was employed to assess short-range attraction, and a dual-choice horizontal olfactometer evaluated host-seeking behaviour. Human odours were obtained from volunteers’ hands, feet, and worn socks. Mosquito responses were analysed for attraction, activity, and inactivity, with statistical analysis performed using two-factor analysis of variance (ANOVA).

**FINDINGS:**

Wild *An. darlingi* mosquitoes showed significantly higher attraction to human odours compared to F1 mosquitoes in both the vertical and horizontal olfactometers. Wild mosquitoes were more attracted to feet and worn socks than F1 mosquitoes, which exhibited low attraction to both stimuli. The preference index (PI) was higher in wild mosquitoes for both hand and sock odours, indicating a stronger attraction to human odours.

**MAIN CONCLUSIONS:**

Wild *An. darlingi* mosquitoes exhibit a stronger behavioural response to human odours compared to F1 mosquitoes. The use of olfactometry, particularly vertical and horizontal methods, proved effective in studying *An. darlingi* mosquito’s host-seeking behaviour and can be applied to further research on vector behaviour and potential control strategies.

The mosquito *Anopheles darlingi* is the primary vector of malaria in the Americas, with a broad distribution spanning several countries in Central America and most of South America, from Colombia and Venezuela to northern Argentina. This species prefers breeding in natural water bodies such as lakes, streams, and slow-flowing rivers. Given these ecological preferences, *An*. *darlingi* thrives in the Amazon region, particularly along forest margins, where environmental conditions support its population persistence.^1^ ^,^ ^2^ ^,^ ^3^ In Brazil, An. darlingi is considered the main vector species involved in the transmission of Plasmodium spp. to humans. This species is highly adaptable to environmental changes such as deforestation and urbanisation.^4^ ^,^ ^5^ Adult *An*. *darlingi* mosquitoes exhibit considerable plasticity in their feeding behaviour, adapting to local environmental conditions and host availability.^6^ They can be found both indoors and outdoors, as well as in peridomestic environments.^7^ Adults exhibit crepuscular-nocturnal habits, during which mating occurs and females perform haematophagy. In the laboratory, specific conditions are required to obtain F1 generation mosquitoes from already inseminated wild females collected in the field. The colonisation of this species required overcoming barriers related to the swarming behaviour for mating and was successfully reported in Brazil in 2019.^8^ Despite its well-established epidemiological importance as a malaria vector, studies investigating *An*. *darlingi*’s attraction to human odour have predominantly relied on baited traps. However, the only study to date that assessed this species’ behaviour using vertical olfactometry with *Plasmodium*-infected patients.^9^

Olfactometry is a well-established technique for studying the host-seeking behaviour of mosquitoes.^10^ ^,^ ^11^ ^,^ ^12^ ^,^ ^13^ It allows researchers to evaluate various behavioural responses, including attraction, activation, and flight pathways in response to chemical (kairomones, *i.e.*, natural or synthetic host odour mimics) and physical (heat, humidity) stimuli.^14^ ^,^ ^15^ Olfactometers have been widely employed to investigate mosquito-host interactions, as well as other behavioural aspects such as feeding,^16^ ^,^ ^17^ ^,^ ^18^ nutrition,^19^ ^,^ ^20^ and reproduction.^21^ ^,^ ^22^

Different olfactometry techniques, including vertical^23^ ^,^ ^24^ and horizontal dual-choice^25^ ^,^ ^26^ methods, can assess mosquito responses to potential host stimuli. Vertical olfactometry evaluates mosquito attraction through passive diffusion of olfactory and physical cues (*e.g.*, humidity and heat) without visual interference.^27^ This method allows the use of human body parts, such as hands or feet, as odour sources, in addition to synthetic attractants. In contrast, horizontal dual-choice olfactometry exposes mosquitoes to a moving odour plume within a controlled airflow system, enabling anaemotaxic responses. This setup facilitates behavioural categorisation into four responses: (a) active (mosquitoes resting or flying outside the choice tube), (b) inactive (mosquitoes remaining in the release cage or failing to take off), (c) attracted (mosquitoes flying against the airflow inside the choice tube), and (d) take-off (mosquitoes leaving the release cage)^28^

This study aimed to assess the feasibility of using vertical and horizontal olfactometry to investigate the behavioural responses of wild *An*. *darlingi* females and first-generation (F1) laboratory-reared females to human odours. These odours were presented through direct stimuli (*e.g.*, hands and feet) and indirect stimuli (*e.g.*, sweat-impregnated synthetic substrates).

## SUBJECTS AND METHODS

*Mosquitoes* - Wild females were collected during the crepuscular-nocturnal period (18:00-22:00 h) by protected human landing catches at three sites: two in Porto Velho (Nova Mutum: 9º18′55.51″S, 64º32′44.96″W; BR 364: 8º49′22.81″S, 63º55′42.57″W) and one in Candeias do Jamari (8º47′31.93″S, 63º42′39.79″W). Approximately 20 specimens were maintained in 500 mL plastic screened cages, placed in insulated boxes, and supplied with 10% sucrose solution on soaked cotton pads. Mosquitoes were transported to the insectary of Laboratório de Bioecologia de Insetos da Universidade Federal de Rondônia, Porto Velho, Brazil. Female An. darlingi were identified using a dichotomous key based on morphological.^29^ ^)^ Insectary conditions were 27 ± 1ºC, 80 ± 5% relative humidity (RH), and a 12:12 h light-dark cycle. Wild females were tested within 48 h of capture, after 24 h of sugar deprivation with access to water only. A subset of wild females was dissected under a stereomicroscope using fine needles and saline solution to verify the presence of spermatozoa in the spermathecae.

As a comparative group, F1 generation females of An. darlingi without previous blood feeding were employed. These were obtained from wild females captured exclusively for colony establishment. Females were fed on Mus musculus (Swiss inbred strain) and, 72 h after feeding, were induced to oviposit by removal of one wing. Resulting larvae were reared under standard insectary conditions (27 ± 1ºC, 80 ± 5% RH, 12:12 h light-dark cycle) and fed ad libitum with fish food (Tetramin Tropical Flakes^®^, Tetra Holding US Inc., USA). Newly emerged F1 females were housed in entomological cages (29.0 × 20.0 × 7.0 cm) and maintained on 10% sucrose solution. Females aged five-seven days were selected as comparative group (F1), representing unfed and unmated females.

Olfactometers

**Fig. 1:**
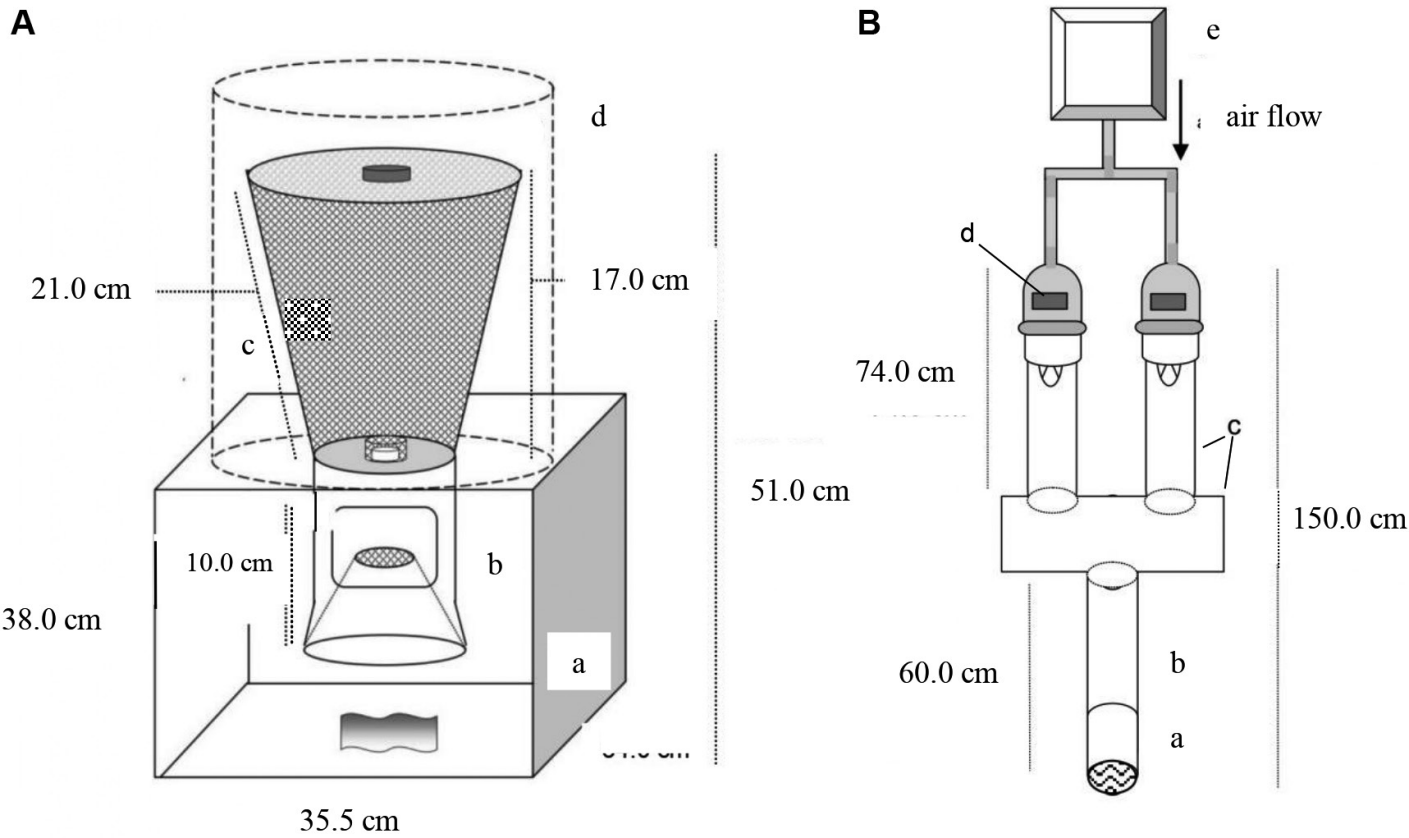
schematic design of experimental olfactometers (A) Vertical olfactometer: (a) open-sided box for stimuli placement; (b) lower cone containing responding insects; (c) screened upper cone for mosquito release; (d) external cylinder to prevent visual interference. (B) Horizontal dual-choice olfactometer: (a) removable release cage; (b) base tube; (c) two choice tubes with stimuli; (d) opening gap to insert stimuli; (e) exhaust pulls air from the outside environment that is filtered through an activated charcoal filter, heated in a water bath generating a 0.30 m/s airflow [27 ± 1ºC, 70% relative humidity (RH) ].

*Vertical olfactometer* - This olfactometer^24^ is composed of four parts, which are made of aluminium to avoid the retention of odour molecules, being (a) a box open on one side (35.5 cm in length, 34.0 cm in width, 37.0 cm height); (b) a cone placed inside the box (10.0 cm in height and 7.0 cm in diameter); (c) a mesh-covered cone positioned on the upper external part of the box (21.0 cm in height, with a top diameter of 14.4 cm and a bottom diameter of 7.4 cm); and (d) a cylinder placed externally around the mesh-covered cone (26.8 cm in height and 22.0 cm in diameter), which is employed to avoid visual interference during tests (Fig. 1A). This type of olfactometer allows the evaluation of chemical (odours) and/or physical stimuli at a short distance through passive diffusion between the cones of the odour plume formed. To do this, the stimuli to be evaluated is inserted below the lower cone and the insects are released at the top of the screened cone and the response to the stimuli offered is measured by the proportion of insects that move from the upper cone to the lower cone. The vertical distance between mosquitoes in the upper cone and the stimuli ranged from 35.05 to 51.0 cm, depending on insect position. Passage from the upper to the lower cone required overcoming a barrier at the cone junction, ensuring that movement was directed by the stimuli.

*Dual-choice olfactometer* - The dual-choice horizontal olfactometer consists of an experimental arena made of acrylic to evaluate the response of mosquitoes to an odour plume. The olfactometer consists of (a) a removable cylindrical release cage (7.0 cm in diameter and 10.0 cm in length, thickness: 0.5 cm) through which mosquitoes were released; (b) an elongated cylindrical base tube (8.0 cm in diameter and 50.0 cm in length) attached to a central rectangular compartment (28,0 cm in length 16.0 cm in width 14.0cm height); (c) two choice tubes (7.0 cm in diameter, 44.0 cm in length) where the stimuli were inserted (10.0 cm in diameter and 20.0 cm in length); and (d) a choice box connected to the three tubes, connected to the three tubes. The odour plume is generated by an air flow, at a speed of 0.30 m/s, drawn by an exhaust (e) fan from the external environment, which is filtered through a heated/humidified charcoal filter (27 ± 1ºC /70% RH) in a water bath and passing the stimuli towards the mosquitoes that are in the release cage (Fig. 1B).

*Selection of volunteers and human odour collection* - Four volunteers (two men and two women, all Caucasian, aged 20-40 years) participated in the study. They were instructed to refrain from using lotions, perfumes, or antiseptic powders on their feet and hands - used as odour sources in the experiments - for four days prior to testing.

Human odour samples were collected using thin black socks made of 100% polyamide (Selene brand). Before use, the socks were washed with a neutral detergent (Extran, 6% concentration: 60 mL/L), dried with hot air (70ºC) for 10 min, and then worn by each volunteer for 10 h until 30 min before testing. The used socks were stored in zip-lock bags (Wyda Zip brand) until the experiments commenced.

Olfactometer bioassays

*Short-distance attractiveness of An. darlingi to human odours using a vertical olfactometer* - To assess short-range attractiveness, experiments were conducted in a vertical olfactometer (modified from Eiras & Jepson)^24^ using wild and F1 *An*. *darlingi* females. Trials began after 6:00 pm, aligning with the mosquitoes’ twilight host-seeking behaviour. Each experiment lasted 10 min, with the first 5 min allowing the mosquitoes to acclimate inside the screened cone before the stimuli was introduced beneath the lower cone. The number of mosquitoes that moved to the lower cone was recorded at the end of each trial.

Two experimental conditions were tested:

• Direct source: One foot of each volunteer served as the odour stimuli (treatment), while an empty olfactometer was employed as the control.

• Indirect source: The stimuli (treatment) was a sock worn by each volunteer, with a clean sock as the control.

Four volunteers participated, and five replicates were performed per stimuli (foot or worn sock) with groups of 10 mosquitoes each, for both wild-type and F1 females. A total of 80 trials were conducted, using 800 females. After each experiment, the olfactometer components were cleaned with a 6% Extran detergent solution (Merck brand) and dried with hot air (70ºC).

*Host-seeking behaviour of An. darlingi in response to human odours using a dual-choice horizontal olfactometer* - Wild and F1 *An*. *darlingi* females were tested in a dual-choice horizontal olfactometer (modified from Paixão et al.)^22^ to evaluate host-seeking behaviour in response to human odours. Trials were conducted after 6:00 pm, beginning with a 10 min acclimatisation period in the release cage, followed by 3 min of exposure to the stimuli introduced into one of the choice tubes.

Two experimental conditions were tested, differing in the stimuli employed:

Experiment 1: A volunteer’s hand was the stimuli (treatment), while the control was an empty choice tube (negative control).

Experiment 2: A worn sock served as the stimuli (treatment), with the control being an empty choice tube (negative control).

Two additional volunteers participated in these experiments. Seven replicates were performed per volunteer per stimuli (hand or worn sock) with groups of 10 mosquitoes each (wild or F1 females). In total, 56 trials were conducted, using 560 mosquitoes.

Following Paixão et al.,^22^ mosquito responses were categorised as:

(a) Attracted: Mosquitoes that entered the choice tubes.

(b) Active: Mosquitoes that left the release cage but did not enter the choice tubes.

(c) Inactive: Mosquitoes that remained in the release cage.

After each experiment, olfactometer components were washed with a 6% Extran detergent solution, air-dried, then cleaned with 70% ethanol and dried again with hot air (70ºC).

*Statistical snalysis* - In vertical olfactometer experiments, the percentage of mosquitoes that responded (*i.e.*, collected in the lower cone) to the stimuli - either the odour of volunteers’ feet or worn socks - was analysed using a two-factor analysis of variance (ANOVA) (mosquito strain × stimuli), followed by Sidak’s post hoc test at a 5% significance level. The control condition was an olfactometer without stimuli.

In dual-choice horizontal olfactometer experiments, the percentage of mosquitoes in each response category - Attracted (entered the treatment arm), Control (entered the control arm), Inactive (remained in the release cage), and Active (left the release cage but did not enter either arm) - was analysed using a two-factor ANOVA (mosquito strain × behaviour), with post hoc comparisons using the Sidak test at a 5% significance level.

The preference index (PI) was calculated using the formula:

$$PI=\frac{\left(Number\;of\;mosquitoes\;in\;the\;treatment\;arm-Number\;of\;mosquitoes\;in\;the\;control\;arm\right)}{\left(Number\;of\;mosquitoes\;in\;the\;treatment\;arm+Number\;of\;mosquitoes\;in\;the\;control\;arm\right)}$$

The PI ranges from 0 to 1, where 0 indicates no preference (neutrality) and 1 indicates maximum attraction. Data were analysed using a two-factor ANOVA (mosquito strain × stimuli), with post hoc comparisons using the Sidak test at a 5% significance level.

*Ethical considerations* - All volunteers were fully informed about the study’s objectives, potential benefits, and risks before providing written informed consent. Ethical approval was granted by the Human Research Ethics Committee of the Research Centre for Tropical Medicine of Rondônia (CAAE No. 41977315200000011).

The use of mice for blood-feeding followed ethical guidelines approved by CEUA/FIOCRUZ-RO (Protocol No. 2014/15).

## RESULTS

**Fig. 2:**
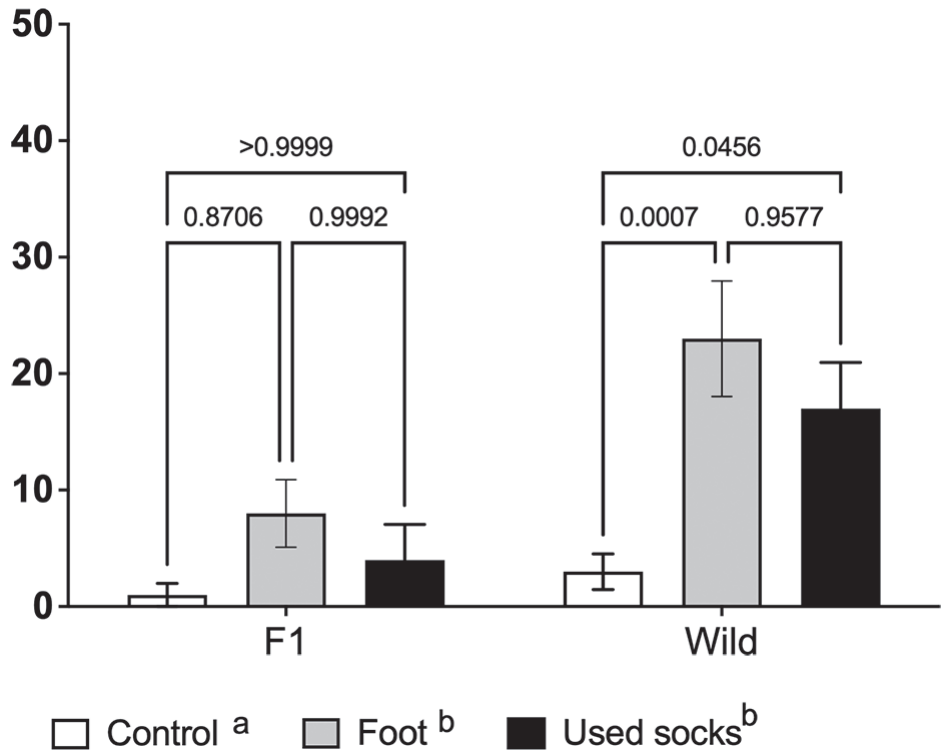
close-range attractiveness of wild and F1 *Anopheles darlingi* to human odours, *i.e.*, feet and used socks, using a vertical olfactometer.

*Assessment of An. darlingi attractiveness to human odours in a vertical olfactometer* - Overall, both feet and used socks attracted more mosquitoes than the empty olfactometer (control) (F = 9.11, p = 0.0004). Wild *An. darlingi* mosquitoes showed a significantly higher attraction to both feet (23%) and used socks (17%) compared to F1 mosquitoes (8% and 4%, respectively) (Feet: F = 7.8, p = 0.0078; Socks: F = 10.9, p = 0.0021). The volunteers’ feet attracted approximately 11 times more wild mosquitoes (23%) than the control condition (empty olfactometer, 2%). In contrast, for F1 mosquitoes, no significant differences were observed between the attraction to feet (8%) and the control (1%), nor between the attraction to used socks (4%) and the control (2%) (p > 0.05) (Fig. 2).

When comparing a used sock versus a clean sock (control) in the same olfactometer, no significant difference was found in the proportion of F1 mosquitoes attracted (p > 0.05). However, for wild mosquitoes, the used sock attracted a significantly higher proportion (16%) compared to the clean sock (5%) (p < 0.05).

**Fig. 3:**
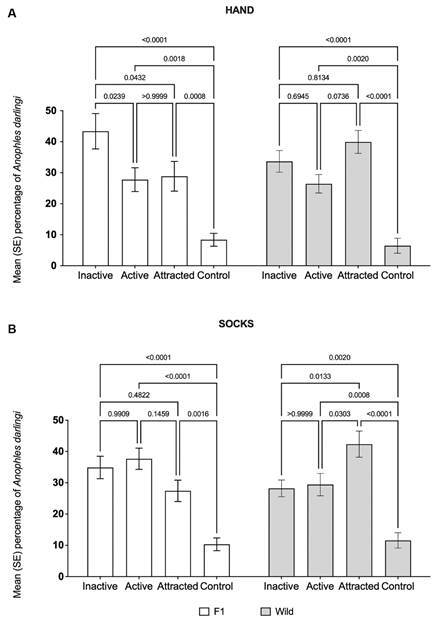
behaviour of wild *Anopheles darlingi* and F1 in a dual-choice horizontal olfactometry assay, using hands and used socks as olfactory stimuli.

*Evaluation of host-seeking behaviour of An. darlingi exposed to human odours using a dual-choice horizontal olfactometer* - F1 mosquitoes showed a significantly higher percentage of inactivity (43.3%) compared to activation (27.8%) when the hand was used as a stimuli (F = 26.3, p < 0.0001). However, no significant differences were observed between activation and inactivity in the other experimental groups and treatments (p > 0.05) (Fig. 3A).

Regardless of the stimuli source or mosquito strain, the percentage of mosquitoes attracted to the stimuli arm was consistently higher than to the control arm.

When hands were employed as stimuli, the attraction rates were 28.8% (F1) and 39.9% (wild-type), while the control arms had significantly lower attraction rates (8.3% for F1, p = 0.0008; 6.5% for wild-type, p < 0.0001).

When used socks were the stimuli, attraction rates were 27.4% (F1) and 42.3% (wild-type), whereas the control arms had 10.3% (F1, p = 0.0016) and 11.6% (wild-type, p < 0.0001) (Fig. 3B).

Inactive: mosquitoes that remained in the release cage. Active: mosquitoes that left the release cage but did not enter the olfactometer arms. Control: mosquitoes attracted to the control arm (without stimuli). Attracted: mosquitoes attracted to the arm with the stimuli.

**Fig. 4:**
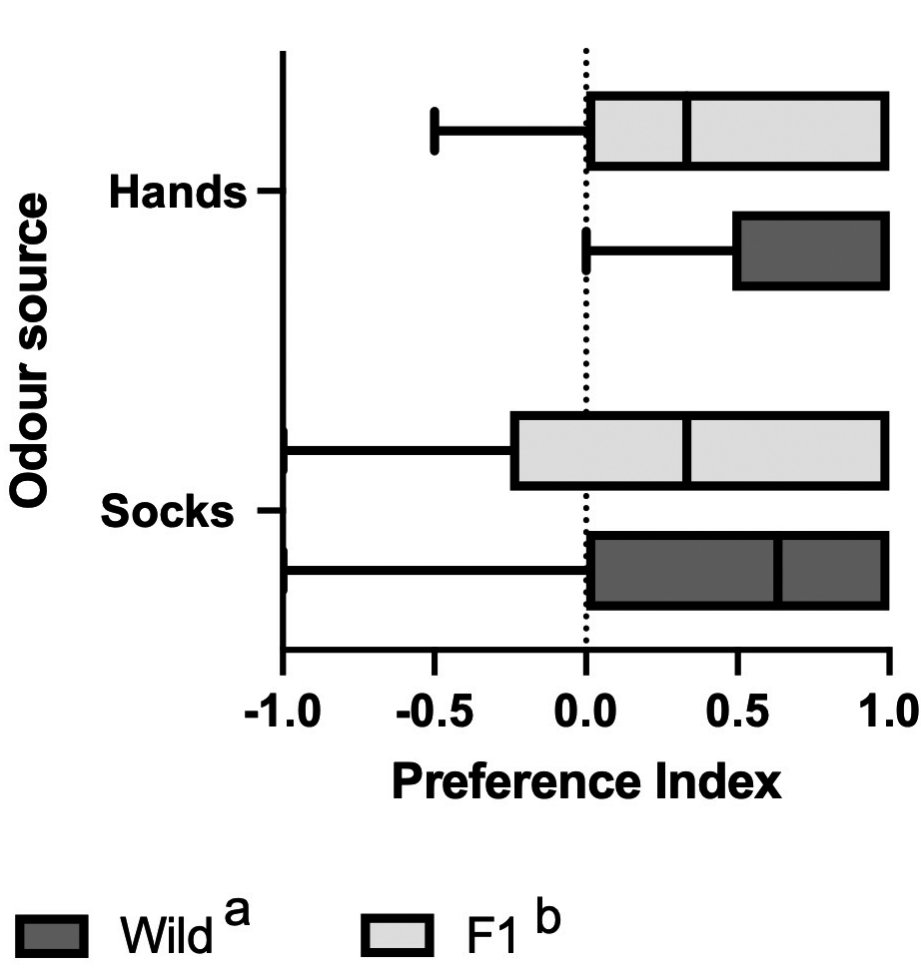
preference index (PI) of wild and F1 *Anopheles darlingi* for hand and used sock odour stimuli from volunteers during dual-choice horizontal olfactometry experiments. Different letters indicate significant differences between treatments (p < 0.05).

The PI of wild *An. darlingi* in dual-choice horizontal olfactometry experiments was significantly higher (F = 6.7; p = 0.01) combining both stimuli - hand and used socks (0.63) - compared to F1 mosquitoes (0.33). However, no significant difference was observed between the responses to hand and sock odours within each mosquito group (F = 3.4; p = 0.07) (Fig. 4).

After examining the spermathecae of a pool of wild An. darlingi females from the collection sites, we found that 93/97 contained sperm, two were empty, and two were lost during dissection.

## DISCUSSION

Olfactometers can be employed to evaluate the oviposition preferences and attraction of *Anopheles* mosquitoes to breeding sites. They also offer the advantage of detecting significant substrates for creating control measures that can modulate the behaviour of gravid females. However, the use of these instruments has some limitations, such as the restricted number of individuals that can be analysed simultaneously and the need for objective quantification methods.^30^

The use of vertical olfactometry with used socks, as well as volunteers’ feet as stimuli in this study, was effective in attracting *An. darlingi* females, but only when wild mosquitoes were employed. The use of sweat-impregnated socks has been successfully applied to attract other mosquito genera, such as *Aedes* and *Culex*.^31^ ^,^ ^32^ However, this is the first successful use of these stimuli for this important Neotropical vector.

In terms of host odour sources, used socks attracted *An. darlingi* females similarly to volunteers’ feet, with no significant differences between these two sources. The results suggest that the used socks effectively captured volatile attractants or components thereof. However, only field-collected females were significantly attracted to the impregnated sock odour sources. The use of socks as an odour source in experiments with *An. darlingi* was effective and supports findings by Okumu et al.^33^ on the effectiveness of synthetic materials (*e.g.*, nylon) for dispersing kairomones in traps for other anopheline species. Additionally, using socks impregnated with odours from patients with symptomatic diseases could enhance research, as patient compliance with travel to laboratories for experiments is often low.^9^ ^,^ ^16^ ^,^ ^33^ In this study, both wild *An. darlingi* females and F1 females were significantly attracted to the odours of the host on impregnated socks and volunteers’ hands (attracted) compared to the control (empty arm). The majority of wild *An. darlingi* females in the present study were inseminated as suggested by our data from a pool of wild females collected in the field. Both inseminated and virgin An. darlingi females are highly active during the scotophase, likely reflecting blood-seeking in the former and mating swarm formation in the latter.^34^ Thus, although both wild and F1 females were expected to be active during the assays, mostly wild (inseminated) females would respond to human odour, consistent with their physiological need for a blood meal to support egg maturation. Similarly, Paixão et al.^22^ reported that both virgin and mated *Ae. aegypti* females were significantly attracted to human hand odours compared to controls (empty cages), although virgin females were generally less attracted than mated females.

Glass spheres impregnated with hand odour were able to attract *An. quadrimaculatus* and *Ae. aegypti* females in a dose-dependent manner in a dual-choice horizontal olfactometer, but were not directly compared to hand odour.^35^ In studies conducted in Kenya, traps baited with used socks collected in huts did not show a difference in capturing *An. gambiae* or *An. funestus* compared to human collection.^11^ Similar results were observed in a study on *An. gambiae* conducted in western Kenya, where no significant difference was found between mosquitoes captured in traps baited with impregnated socks and human odour.^36^ However, individual differences in volatile profiles, including between sexes, should not be disregarded.^37^

Host-seeking behaviour in female Anopheles mosquitoes is strongly modulated by their physiological state. Age, body size, nutritional reserves, and gonotrophic status all influence the likelihood and intensity of host-seeking responses.^38^ ^,^ ^39^ ^,^ ^40^ Importantly, some Anopheles species exhibit opportunistic host seeking even before mating, indicating that reproductive and nutritional demands may overlap during the early adult phase.^41^ Thus, the responsiveness of females to semiochemicals such as CO₂ and human-derived volatiles is not fixed, but varies dynamically according to their physiological condition.^42^ ^,^ ^43^

In our comparison of the two olfactometer designs, it is important to note that the vertical olfactometer relies on passive diffusion of host odour, which results in slower accumulation of volatiles and delayed mosquito responses, often taking several minutes after the stimuli is introduced. In contrast, dual-port horizontal olfactometers employ a forced and filtered airflow that continuously delivers host odours in a controlled manner, producing a more uniform odour plume and eliciting rapid, upwind flight responses.^15^ Therefore, it is expected that response rates in the horizontal olfactometer will generally exceed those obtained in the vertical design, as the former better reproduces the ecological conditions under which mosquitoes locate their hosts.

Our findings demonstrate that wild An. darlingi females exhibited significantly stronger behavioural responses to human odours than laboratory-reared F1 virgins, highlighting the influence of physiological state on host-seeking activity. Both vertical and horizontal olfactometers proved to be effective tools for assessing mosquito attraction to direct (hands, feet) and indirect (worn socks) human odour stimuli, except in the vertical olfactometer using F1 mosquitoes. Worn socks performed comparably to human feet, supporting their use as standardised odour sources in laboratory assays. Overall, olfactometry offers a valuable approach for advancing behavioural studies of An. darlingi and provides insights that may contribute to the development of odour-based monitoring and control strategies for this malaria vector.

## Data Availability

The contents underlying the research text are included in the manuscript.
